# Natural Compounds Targeting Thymic Stromal Lymphopoietin (TSLP): A Promising Therapeutic Strategy for Atopic Dermatitis

**DOI:** 10.3390/biom14121521

**Published:** 2024-11-27

**Authors:** Muhammad Suleman, Chiara Moltrasio, Paola Maura Tricarico, Angelo Valerio Marzano, Sergio Crovella

**Affiliations:** 1 Laboratory of Animal Research Center (LARC), Qatar University, Doha 2713, Qatar; m.suleman@qu.edu.qa; 2 Dermatology Unit, Fondazione IRCCS Ca’ Granda Ospedale Maggiore Policlinico, 20122 Milan, Italy; chiara.moltrasio@policlinico.mi.it (C.M.); angelo.marzano@unimi.it (A.V.M.); 3 Department of Pediatrics, Institute for Maternal and Child Health—IRCCS Burlo Garofolo, 34137 Trieste, Italy; paolamaura.tricarico@burlo.trieste.it; 4 Department of Pathophysiology and Transplantation, Università degli Studi di Milano, 20122 Milan, Italy

**Keywords:** atopic dermatitis, TSLP, molecular docking, MD simulation, binding free energies

## Abstract

Atopic dermatitis (AD) is a chronic inflammatory skin disease with rising prevalence, marked by eczematous lesions, itching, and a weakened skin barrier often tied to filaggrin gene mutations. This breakdown allows allergen and microbe entry, with thymic stromal lymphopoietin (TSLP) playing a crucial role by activating immune pathways that amplify the allergic response. TSLP’s central role in AD pathogenesis makes it a promising therapeutic target. Consequently, in this study, we used the virtual drug screening, molecular dynamics simulation, and binding free energies calculation approaches to explore the African Natural Product Database against the TSLP protein. The molecular screening identified four compounds with high docking scores, namely SA_0090 (−7.37), EA_0131 (−7.10), NA_0018 (−7.03), and WA_0006 (−6.99 kcal/mol). Furthermore, the KD analysis showed a strong binding affinity of these compounds with TSLP, with values of −5.36, −5.36, −5.34, and −5.32 kcal/mol, respectively. Moreover, the strong binding affinity of these compounds was further validated by molecular dynamic simulation analysis, which revealed that the WA_0006-TSLP is the most stable complex with the lowest average RMSD. However, the total binding free energies were −40.5602, −41.0967, −27.3293, and −51.3496 kcal/mol, respectively, showing the strong interaction between the selected compounds and TSLP. Likewise, these compounds showed excellent pharmacokinetics characteristics. In conclusion, this integrative approach provides a foundation for the development of safe and effective treatments for AD, potentially offering relief to millions of patients worldwide.

## 1. Introduction

Atopic dermatitis (AD) is a common chronic inflammatory skin condition characterized by eczematous lesions, intense itching, and xerosis, significantly affecting the quality of life of millions worldwide. AD’s prevalence has risen dramatically in recent decades, particularly in industrialized nations, and its pathogenesis is complex, involving both genetic and environmental factors. A key feature of AD is the breakdown of the epidermal barrier, often linked to mutations in the filaggrin gene, which facilitates the penetration of allergens and microbes into the skin [1]. This results in an abnormal immune response dominated by T helper type 2 (Th2) cells, which produce interleukins that further exacerbate the inflammation [2]. Thymic stromal lymphopoietin (TSLP), a cytokine produced primarily by epithelial cells, has emerged as a critical player in this process. TSLP is highly expressed in the lesional skin of AD patients and acts by activating dendritic cells, which then promote the differentiation of naive T cells into Th2 cells, amplifying the allergic response [3]. Furthermore, TSLP influences the activity of other immune cells, such as basophils, eosinophils, and innate lymphoid cells, all of which contribute to the chronic inflammation characteristic of AD [4]. Given the central role of TSLP in the pathogenesis of AD, it has become a key target for therapeutic interventions. Indeed, therapies that block TSLP signaling, such as biologics, have shown promise in reducing AD symptoms [5]. However, these treatments can be costly and may have long-term side effects, highlighting the need for safer, more accessible alternatives [6]. In this context, natural compounds have gained increasing attention due to their broad range of bioactive properties, low toxicity, and potential to modulate key pathways involved in AD, including TSLP signaling [7]. Several natural compounds, such as quercetin, curcumin, epigallocatechin gallate (EGCG), licorice (Glycyrrhiza glabra), resveratrol, and Centella asiatica, have been shown to regulate the expression of TSLP and other pro-inflammatory mediators [8,9]. Quercetin, a flavonoid found in many fruits and vegetables, has been reported to inhibit TSLP secretion in keratinocytes, thereby reducing the activation of dendritic cells and the downstream Th2 response [10]. Similarly, curcumin, the active compound in turmeric, has demonstrated the ability to suppress TSLP expression in various inflammatory models, including AD, by modulating nuclear factor kappa B (NF-κB) signaling [11]. EGCG, the major catechin in green tea, has been shown to reduce TSLP levels in allergic skin inflammation models and inhibit the activation of dendritic cells [12]. Licorice root extract, particularly its active component glycyrrhizin, has long been used in traditional medicine for its anti-inflammatory properties and has recently been found to downregulate TSLP expression in keratinocytes, making it a potential candidate for AD treatment [13]. Resveratrol, a polyphenol found in grapes and berries, has also been shown to inhibit TSLP production and attenuate Th2-mediated inflammation in AD models [14]. Centella asiatica, commonly known as Gotu Kola, is a traditional medicinal herb that has demonstrated significant anti-inflammatory effects, including the inhibition of TSLP production in human keratinocytes [15]. These natural compounds not only reduce TSLP expression but also target other aspects of the immune response, providing a multifaceted approach to AD management. Despite the promising results from preclinical studies, further research is needed to validate the clinical efficacy of these natural compounds and determine optimal formulations and dosages for AD treatment. Moreover, the potential for synergistic effects between these compounds and existing therapies, such as corticosteroids and biologics, warrants exploration. As the demand for more natural and holistic approaches to treating chronic diseases like AD grows, the development of TSLP-targeted therapies using natural compounds offers a promising avenue for future research and clinical practice. This study demonstrates the potential of using of virtual drug screening and molecular dynamics simulations to reposition natural compounds for the treatment of atopic dermatitis. In this study, the African natural compounds were screened against TSLP, and strong binding affinity of the selected compounds was further verified by using the molecular dynamics simulation and binding free calculation approaches. The integrative approach adopted herein not only enhances our understanding of the molecular interactions involved but also provides a foundation for the development of safer and more effective treatments for AD, potentially offering relief to millions of patients worldwide.

## 2. Materials and Methods

### 2.1. Crystal Structure Retrieval and Preparation

The crystal structure of the TSLP complex (PDB ID: 5J12) was sourced from the Protein Data Bank (RCSB PDB) [16] (https://www.rcsb.org/structure/5nt1) (accessed on 7 August 2024). PyMOL was then used to remove any water molecules from the structure [17]. Hydrogen atoms were added, and structural minimization was performed on the protein using Chimera [18,19].

### 2.2. Molecular Screening of Natural Product Libraries

This study utilized the African Natural Product Database, containing diverse African medicinal compounds, and screened them for drug-likeness and toxicity using the FAF4drug webserver, filtering out compounds that violated Lipinski’s rule of five [19]. Ligands were prepared by converting them to the pdbqt format, with tools like Open Babel assigning charges and atom types; the ligand preparation process included considering non-polar hydrogen atoms, Gasteiger charges, and defining torsion tree roots for flexibility analysis, while receptors were prepared using AutoGrid to define the docking space. The virtual screening was performed with EasyDock Vina 2.0, starting with an initial low-exhaustiveness screening (set to 16) for efficiency, followed by a more rigorous re-screening (exhaustiveness of 64) for top hits to reduce false positives. Then, the top 10% of high-ranking compounds underwent induced-fit docking (IFD) with AutoDockFR, accommodating receptor flexibility and enabling covalent docking using default parameters [20]. AutoDockFR generally utilizes force fields like AMBER or CHARMM, molecular dynamics (MD) simulation protocols, and scoring functions such as AMBER scoring or force-field-based scoring for induced-fit docking (IFD) simulations. Finally, on the basis of high docking scores and favorable interaction, the top four compounds were selected and virtualized with PyMOL software [21].

### 2.3. Molecular Simulation of Shortlisted Drug–Protein Complexes

The molecular dynamics (MD) simulations were set up using AMBER21, a robust tool recognized for its efficient algorithms and high precision, giving researchers detailed insight into biomolecular interactions. Using the tLeap module, coordinates and topology files for each protein–ligand complex were generated [22,23]. Each system was immersed in an Optimal Point Charge (OPC) water box, and counterions were added to neutralize any net charges. Ligand parameters were derived using the GAFF2 force field, with initial topology and frcmod files created via antechamber and parmchk2 tools. Energy minimization was carried out iteratively, alternating between steepest descent and conjugate gradient methods, until a specified threshold for energy change or force was reached. Systems were then gradually heated to the target temperature, with temperature controlled using coupling algorithms such as Langevin dynamics or the Berendsen thermostat. Long-range electrostatics were computed using the Particle Mesh Ewald (PME) approach, and van der Waals forces were modeled with the Lennard-Jones potential [24]. Equilibration occurred in stages, involving restrained minimization, controlled heating, and unrestrained stabilization to reach stable conditions at the designated temperature and pressure. The SHAKE algorithm ensured fixed covalent bond lengths and angles, while pressure control was managed through a barostat (Berendsen or Andersen) [25]. Each system then proceeded to a 300-nanosecond production simulation under either NPT or NVT ensemble conditions [26].

### 2.4. Post-Simulation Analysis of the Top Hit-NS1 Complexes

The trajectory generated from the simulation production was evaluated using the CPPTRAJ or PTRAJ modules [27]. This analysis involved computing several critical metrics, including RMSD, RMSF, radius of gyration (Rg), and hydrogen bonding interactions for each system [28,29,30]. RMSD can be calculated using the equation outlined below:

(1) $$\mathrm{RMSD}=\sqrt{\frac{\sum d^{2}i=1}{N_{atoms}}}$$

Here, ‘di’ denotes the positional variance between atoms, while ‘i’ refers to both the original and superimposed structures. Moreover, to analyze the fluctuation of the TSLP protein after binding to the ligand molecule, we calculated the RMSF by using the following mathematical equation:

(2) $$Thermal\;factor\;or\;B\text{-}factor=\left[\left(8\pi **2\right)/3\right]\;\left(msf\right)$$

However, to analyze the compactness of the shortlisted compounds and TSLP complexes, we calculated the Rg value with respect to time by using the following equation:

(3) $$r_{RG}^{2}=\frac{\sum_{i=1}^{N}m_{i}{\left(r_{i}-r_{CM}\right)}^{2}}{\sum_{i=1}^{N}m_{i}}$$

### 2.5. Binding Free Energy and Dissociation Constant (KD) Analysis

The total binding free energy at the end-point was assessed using the molecular mechanics generalized Born surface area (MM/GBSA) methodology, a well-established and dependable technique for determining the binding affinity of protein-ligand complexes [21]. To perform the free energy calculations, stable frames from the simulation trajectories were selected utilizing the MMPBSA.py script [19]. The following equations were used to calculate the total binding free energies for the shortlisted compounds and TSLP target protein:

(4) $$\Delta G_{bind}=G_{\left(complex,\;\;\;solvated\right)}-G_{\left(receptor,\;\;\;solvated\right)}-G_{\left(ligand\;\;\;solvated\right)}$$

This formula is used to evaluate the role or impact of interactions within the complex and can be expressed as follows:

(5) $$G=E_{Molecular\;Mechanics}-G_{solvated}-TS$$

The rearranged versions of the above equation for calculating specific energy are as follows:

(6) $$\Delta G_{bind}=\Delta E_{Molecular\;Mechanics}+\Delta G_{solvated}-\Delta TS=\Delta G_{vaccum}+\Delta G_{solvated}$$

(7) $$\Delta E_{Molecular\;Mechanics}=\Delta E_{int}+\Delta E_{electrostatic}+\Delta E_{vdW}$$

(8) $$\Delta G_{solvated}=\Delta G_{Generalized\;born}+\Delta G_{surface\;area}$$

(9) $$\Delta G_{surface\;area}=\gamma .SASA+b$$

(10) $$\Delta G_{vaccum}=\Delta E_{Molecular\;Mechanics}-T\Delta S$$

Furthermore, the dissociation constant (KD) for the top hits was computationally predicted by using PRODIGY-LIGAND webservers. This server was previously used to predict the KD of different molecules used against different diseases [31].

### 2.6. Lipinski’s Rule and Pharmacokinetics Analysis

Lipinski’s rule of five is a critical framework in drug development, indicating that effective orally administered drugs typically exhibit certain molecular characteristics. These include a molecular weight of less than 500, a maximum of five hydrogen bond donors, no more than ten hydrogen bond acceptors, and a logP value of five or lower. This guideline helps predict a compound’s oral bioavailability and permeability, thereby optimizing its drug-like properties for successful pharmaceutical applications [32]. To evaluate adherence to these criteria, we utilized the online platform SwissADME (http://www.swissadme.ch/) (accessed on 15 September 2024) [33]. Furthermore, the assessment of ADMET (absorption, distribution, metabolism, excretion, and toxicity) properties is essential, as nearly 50% of drug candidates fail during development due to non-compliance with these pharmacokinetic principles [34]. In silico ADMET analyses were performed using the pkCSM tool (https://biosig.lab.uq.edu.au/pkcsm/) (accessed on 15 September 2024), where we calculated key pharmacokinetic parameters [35]. These included water solubility, Caco-2 permeability, human intestinal absorption, blood–brain barrier penetration, cytochrome P450 inhibition and substrate potential, AMES toxicity, skin sensitization, and hepatotoxicity for the top-selected natural compounds.

## 3. Results and Discussion

### 3.1. Virtual Drug Screening of Phytocompounds Against the TSLP Protein

Virtual drug screening plays a vital role in the drug design field by expediting the identification of promising drug candidates and enhancing their chemical and biological properties. Virtual drug screening of phytocompounds offers a fast, cost-effective way to explore natural compounds for new therapeutics, making it invaluable in drug discovery. By screening large libraries of plant-derived molecules, researchers can identify novel drug candidates with unique structural features that are often safer and more effective. This approach targets specific disease-related biomolecules, allowing for more precise and potent therapies. Additionally, virtual screening reduces environmental impact, enables toxicity and drug-likeness predictions, and accelerates lead optimization, helping prioritize compounds with the highest therapeutic potential. Overall, it bridges traditional natural medicine and advanced technology, facilitating the development of affordable and targeted treatments for complex diseases [36,37,38,39]. Consequently, in the present study, we used the virtual drug screening of the South African Natural Product Database to identify the lead compounds that can target the TSLP protein. Before conducting database screening, we applied Lipinski’s rule of five to filter out drug-like molecules, which has been used by several studies [40]. The computational drug screening was performed using AutoDock Vina, targeting the TSLP protein. We initially evaluated 954 compounds from the South African database, narrowing down to 793 based on adherence to the rule of five. These selected compounds, totaling 823, then entered a multi-tiered screening process. In the first stage of virtual screening, docking scores ranged from −5.72 to 4.49 kcal/mol, allowing us to focus on compounds scoring between −5.72 and −5.50 kcal/mol. This subset was then processed through induced-fit docking, where scores fell between −7.37 and −6.50 kcal/mol. From this group, only 10 compounds stood out, displaying docking scores above −6.80 kcal/mol alongside strong interaction profiles. Based on the high docking score, only four compounds, namely 8-oxo-16-[(2R,3S,4S,5S,6R)-3,4,5-trihydroxy-6-(hydroxymethyl)tetrahydropyran-2-yl]oxy-hexadecanoic, [(2S,4aS)-5-hydroxy-7-isopropyl-1,1,4a-trimethyl-6-oxo-3,4-dihydro-2H-phenanthren-2-yl], (2R,3S,4R,5R)-2,3,4,5-tetrahydroxy-6-[(2R)-5-hydroxy-2-(4-methoxyphenyl)-4-oxo-chroman-7-yl]oxy-hexa, and (2S,3S)-6-[(2E)-3,7-dimethylocta-2,6-dienyl]-3,5,7-trihydroxy-2-(4-hydroxyphenyl)chroman-4-one, with docking scores of −7.37 kcal/mol, −7.10 kcal/mol, −7.03 kcal/mol, and −6.99 kcal/mol, respectively, were selected for further analysis. We are also including the compounds’ IUPAC equivalents to ensure both scientific accuracy and practical utility. These are, respectively, (2R,3S,4S,5S,6R)-2-[16-(8-oxohexadecanoyl)oxy]-3,4,5-trihydroxy-6-(hydroxymethyl)tetrahydropyran, 2S,4aS)-5-hydroxy-7-isopropyl-1,1,4a-trimethyl-6-oxo-1,2,3,4-tetrahydrophenanthrene, (2R,3S,4R,5R,6S)-6-[(2R)-5-hydroxy-2-(4-methoxyphenyl)-4-oxo-4H-1-benzopyran-7-yloxy]-2,3,4,5-tetrahydroxyhexanal, and (2S,3S)-2-(4-hydroxyphenyl)-3,5,7-trihydroxy-6-[(2E)-3,7-dimethylocta-2,6-dienyl]-4H-1-benzopyran-4-one.

The drugs’ IDs, 2D structures, name, and docking score are shown in Table 1. 

### 3.2. Bonding Network Analysis of Shortlisted Compounds and TSLP Complexes

**Figure 1:**
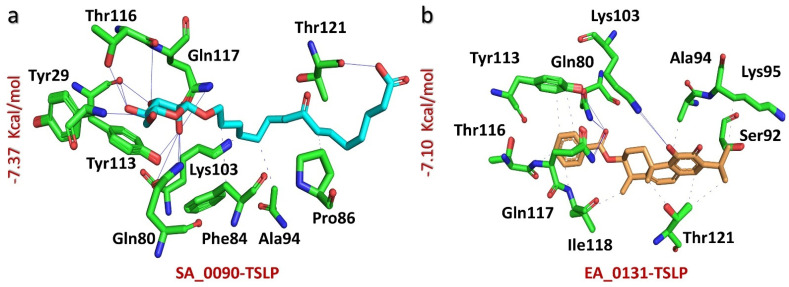
Bonding network analysis of SA_0090–TSLP and EA_0131-TSLP complexes. (**a**) shows the stick representation of the bonding network of the SA_0090-TSLP complex. (**b**) shows the stick representation of the bonding network of the EA_0131-TSLP complex.

The interaction analysis of TSLP complexes with compounds SA_0090 and EA_0131 uncovered a variety of stabilizing forces, including both hydrogen bonds and hydrophobic interactions, between each compound and the protein target. For the SA_0090-TSLP complex, a docking score of −7.37 kcal/mol indicated strong binding affinity, which was reinforced by 11 hydrogen bonds and four hydrophobic contacts with key amino acids. Notable residues contributing to these interactions were Tyr29, Gln80, Phe84, Pro86, Ala94, Lys103, Tyr113, Thr116, Gln117, and Thr121 (Figure 1a). Similarly, the EA_0131-TSLP complex achieved a docking score of −7.10 kcal/mol, attributed to seven hydrogen bonds and 13 hydrophobic contacts. Essential residues facilitating these interactions included Gln80, Ser92, Ala94, Lys95, Lys103, Tyr113, Thr116, Gln117, Ile118, and Thr121, supporting both hydrogen bonding and hydrophobic interactions (Figure 1b). These results emphasize the crucial role of specific residues in promoting stable and effective drug-protein complexes. 

**Figure 2:**
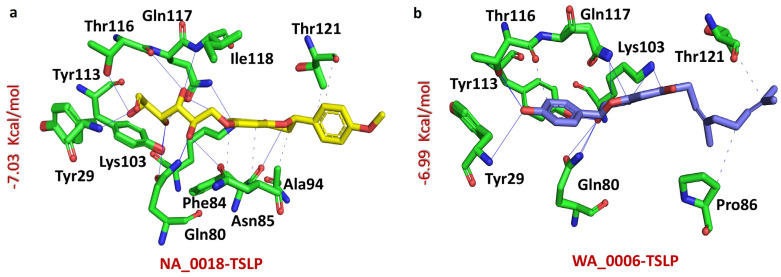
Bonding network analysis of NA_0018-TSLP and WA_0006-TSLP complexes. (**a**) shows the stick representation of the bonding network of the NA_0018-TSLP complex. (**b**) shows the stick representation of the bonding network of the WA_0006-TSLP complex.

Moreover, NA_0018 compound displayed a significant affinity for the TSLP protein, achieving a docking score of −7.03 kcal/mol. This interaction involved a dense network of bonds, forming 14 hydrogen bonds and five hydrophobic contacts. Key residues involved included Tyr29, Gln80, Phe84, Asn85, Ala94, Lys103, Tyr113, Thr116, Gln117, Ile118, and Thr121 (Figure 2a). On the other hand, the WA_0006-TSLP complex showed a binding score of −6.99 kcal/mol, facilitated by 12 hydrogen bonds and three hydrophobic interactions with residues such as Tyr29, Gln80, Pro86, Lys103, Tyr113, Thr116, Gln117, and Thr121 (Figure 2b). These results highlight the potential of these compounds as effective TSLP-targeting agents, as each demonstrates stable interactions, suggesting their promising therapeutic utility.

### 3.3. Dissociation Constant (KD) Analysis of Shortlisted Compound-TSLP Complexes

KD analysis is essential in protein–drug interactions because it quantifies the binding affinity between a drug and its target, guiding key aspects of drug development [41,42]. A low KD indicates high affinity, suggesting the drug binds strongly to its target, which can improve therapeutic efficacy, specificity, and reduce off-target effects. This insight is crucial for drug optimization, as it helps identify compounds with favorable binding properties early on, supports dose determination, and aids in predicting biological activity [43,44]. Additionally, KD data help elucidate a drug’s mechanism of action and drive structure–activity relationship (SAR) studies, enabling fine-tuning of molecular structures for enhanced potency and safety in therapeutic applications. Consequently, to check the binding affinity of the shortlisted compounds with the TSLP protein, we calculated the dissociation constant by using the PRODIGY-LIGAND webservers [45]. The analysis revealed a KD value of −5.36 kcal/mol for the SA_0090–TSLP complex, −5.34 kcal/mol for the EA_0131-TSLP and NA_0018-TSLP complexes, and −5.32 kcal/mol for the WA_0006-TSLP complex. A similar range of values has been previously reported for the strong binding affinity of drugs with the target proteins [31,46]. This analysis further verifies the molecular docking results. 

### 3.4. Molecular Dynamics Simulation of Shortlisted Compound Complexes

**Figure 3:**
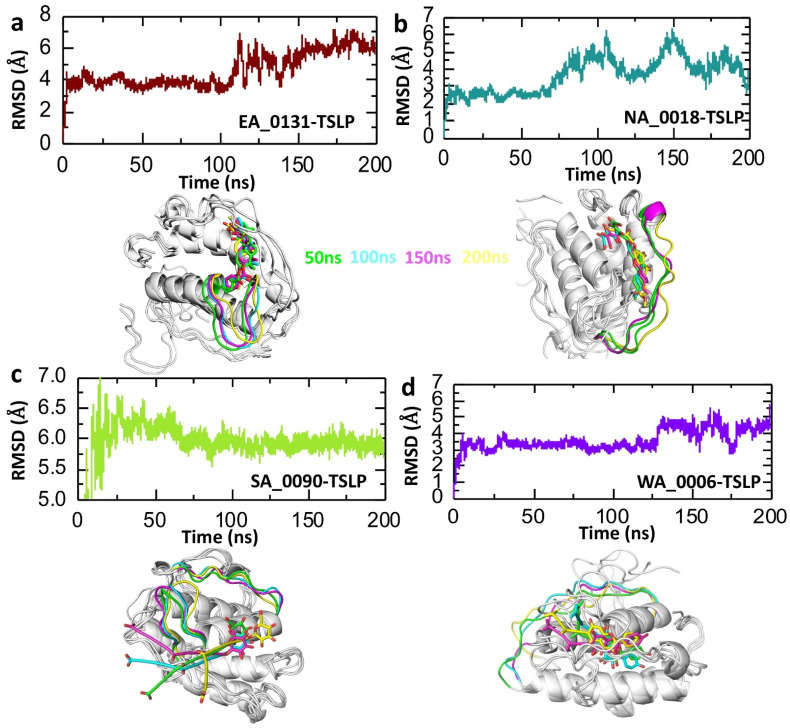
Dynamic stability analysis of shortlisted drug-TSLP complexes. (**a**) The trajectories of the RMSD for the EA_0131-TSLP complex over time. (**b**) The trajectories of the RMSD for the NA_0018-TSLP complex over time. (**c**) The trajectories of the RMSD for the SA_0090-TSLP complex over time. (**d**) The trajectories of the RMSD for the WA_0006-TSLP complex over time.

Root Mean Square Deviation (RMSD) is a vital parameter in molecular dynamics (MD) simulations, commonly used to analyze the structural stability and conformational changes of biomolecules, such as protein-ligand complexes, over time. For drug–protein interactions, RMSD values reflect how much the complex deviates from its initial structure as the simulation progresses. A low and stable RMSD indicates a strong, stable interaction, suggesting that the ligand fits well within the binding site of the protein without causing significant structural perturbations [47]. Conversely, high or fluctuating RMSD values may indicate an unstable interaction or flexibility within the complex, which could suggest a weaker binding affinity or substantial conformational adjustments [48,49]. Consequently, to check the stability of our shortlisted compounds with the TSLP protein, we calculated the RMSD with respect to time. Figure 3 shows the RMSD plots, with the temporal stability and dynamics of four different protein–ligand complexes, labeled as EA_0131-TSLP, NA_0018-TSLP, SA_0090-TSLP, and WA_0006-TSLP. Each plot represents the RMSD in angstroms (Å) over a 200-nanosecond (ns) simulation. In the case of the EA_0131-TSLP complex, the RMSD initially stabilized around 4 Å within the first 20 ns and remained stable until 100 ns with high convergence. After this point, the RMSD value suddenly increased to 6 Å, which was maintained until the end of the simulation with moderate fluctuations. The average for this system was found to be 4.5 Å. The stability of the ligand within the binding cavity was assessed by superimposing structures obtained from various simulation time points (50 ns, 100 ns, 150 ns, and 200 ns). This comparison demonstrated consistent alignment, indicating that the ligand maintained a stable position throughout the simulation (Figure 3a). However, in the case of the NA_0018-TSLP system, higher stability was observed initially with a 2.5 Å RMSD. The value of the RMSD increased to 5 Å after 80 ns and then remind stable until the end of the simulation, with an average RMSD of 3.7 Å. The superimposed structures further verify the stability; however, fluctuation in the RMSD value is due to the loop region around the ligand binding cavity (Figure 3b). Moreover, in the case of SA_0090-TSLP, the RMSD initially fluctuated significantly for short period of time and then stabilized at around 6 Å, and it remained relatively stable near this value with a high convergence throughout the simulation. The average RMSD for this system was 6 Å with the table interaction of the ligand in the binding cavity as shown by superimposing the structures retrieved at different time points of the simulation (Figure 3c). This indicates a stable interaction, albeit with a higher average deviation compared to the EA_0131-TSLP and NA_0018-TSLP complexes. The consistency in RMSD suggests that the SA_0090 ligand fits well into the binding pocket, maintaining a stable interaction without major structural changes in the complex. In contrast, the WA_0006-TSLP complex showed the lowest RMSD among the four, stabilizing at around 3.5 Å after an initial rise in the first few nanoseconds. This stability is maintained with minimal fluctuations, indicating a very stable interaction. The low and consistent RMSD suggests that the WA_0006 ligand forms a strong and stable binding with TSLP, with minimal conformational adjustments required over the course of the simulation. Furthermore, the superimposed structure retrieved at different time points further verified that the ligand was stably attached to the cavity throughout the simulation time period (Figure 3d). In summary, WA_0006-TSLP is the most stable complex with the lowest average RMSD, suggesting a stronger and more stable interaction. NA_0018-TSLP (b) is the least stable, showing considerable structural flexibility. 

**Figure 4:**
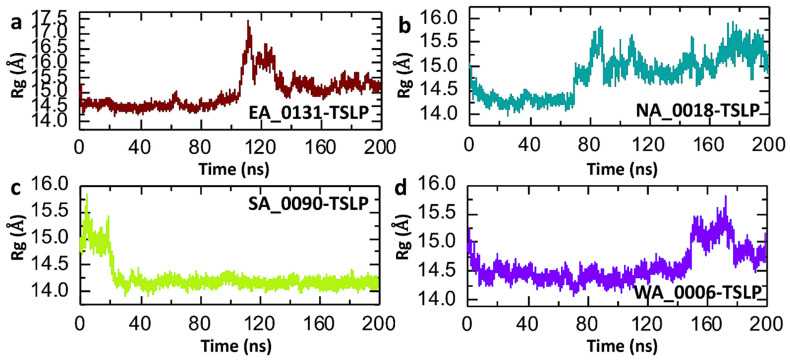
Post-simulation trajectory analysis for residual compactness of shortlisted compound-TSLP complexes. (**a**) represents the compactness of the EA_0131-TSLP complex. (**b**) represents the compactness of the NA_0018-TSLP complex. (**c**) represents the compactness of the SA_0090-TSLP complex. (**d**) represents the compactness of the WA_0006-TSLP complex.

The radius of gyration (Rg) is a measure of the compactness of a protein or protein–ligand complex during molecular dynamics (MD) simulations. It reflects the distribution of the atoms around the center of mass of the system and is an important parameter to assess the structural stability and folding behavior of the complex [50,51]. A stable Rg over time suggests a compact and stable structure, while significant fluctuations can indicate conformational changes, expansion, or potential unfolding of the protein or complex [52]. Consequently, to check the compactness of the shortlisted compound-TSLP complexes, we calculated the Rg with respect to time. In the case of the EA_0131-TSLP complex in the initial 80 ns, the Rg value remains relatively stable at around 14.0–14.5 Å, indicating a compact and stable structure during this period. However, at approximately 100 ns, there is a noticeable increase in Rg to around 17.0 Å, suggesting that the complex undergoes a conformational expansion or loosening. After this peak, the Rg decreases but remains above 14.5 Å, showing moderate fluctuations for the remainder of the simulation. This indicates that the complex does not fully revert to its initial compact structure, suggesting partial structural rearrangements that stabilize at a new level (Figure 4a). Furthermore, in the case of the NA_0018-TSLP complex, a similar pattern of Rg was observed as found in the RMSD plot. Initially, the plot shows a stable Rg value until 65 ns, and then a gradual increase in Rg was recorded, starting from around 14.0 Å and reaching nearly 15.5 Å towards the end. The consistent increase indicates a gradual expansion of the complex, which may reflect a lack of compactness and stability. The fluctuations in the Rg values towards the end of the simulation suggest some dynamic structural rearrangements that prevent the complex from achieving a compact form (Figure 4b). Moreover, the Rg for the SA_0090-TSLP complex quickly stabilizes at around 14.0 Å after an initial adjustment, and it remains consistent with no fluctuations throughout the 200 ns simulation. This stable Rg value suggests that the SA_0090-TSLP complex maintains a compact and stable structure without significant conformational changes. The SA_0090-TSLP complex is highly stable, showing minimal fluctuations in Rg, indicating that the complex remains tightly packed and structurally sound over the course of the simulation (Figure 4c). Furthermore, the Rg of the WA_0006-TSLP complex is initially stable at around 14.5 Å and remains relatively stable with no fluctuations throughout the simulation. There is a small increase in Rg towards the end of the simulation, reaching around 15.0 Å, but the changes are minor and do not indicate significant structural expansion. The WA_0006-TSLP complex demonstrates overall stability with only minor fluctuations, suggesting that the complex is compact and stable for most of the simulation, with a slight tendency towards expansion near the end (Figure 4d). This analysis of the Rg plots complements the previous RMSD analysis, confirming that SA_0090-TSLP and WA_0006-TSLP are the most stable complexes, while NA_0018-TSLP is likely unstable and may not form a strong, compact interaction with the TSLP protein.

**Figure 5:**
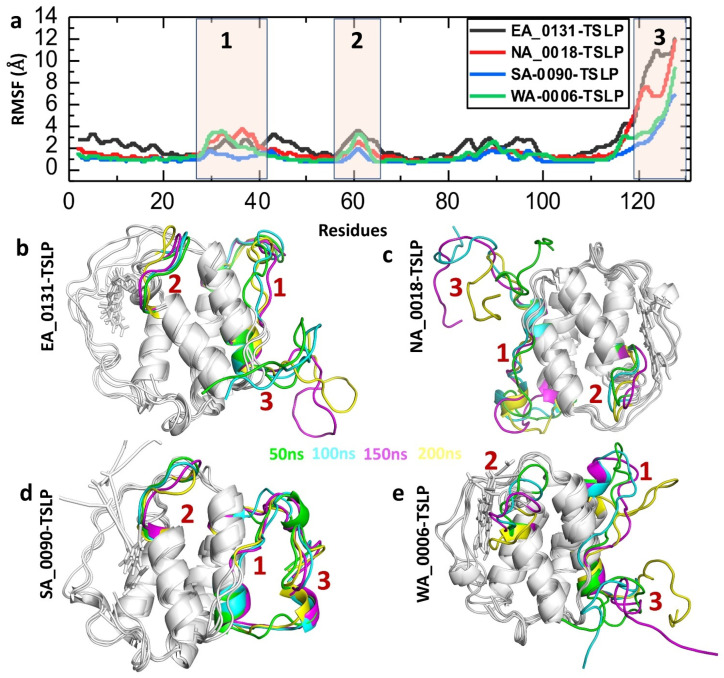
(**a**) Post-simulation trajectory analysis for the RMSF of shortlisted compound-TSLP complexes. Note: the different colors show the specific compounds. (**b**) Showing the fluctuating regions of EA_0131-TSLP complex. (**c**) Showing the fluctuating regions of NA_0018-TSLP complex. (**d**) Showing the fluctuating regions of SA_0090-TSLP complex. (**e**) Showing the fluctuating regions of WA_0006-TSLP complex.

The Root Mean Square Fluctuation (RMSF) provides insight into the flexibility of each residue in a protein or protein–ligand complex over the course of a molecular dynamics (MD) simulation [53]. High RMSF values for specific residues indicate regions with significant movement or flexibility, whereas low RMSF values indicate rigid or stable regions [54]. Therefore, to check the residual fluctuation of TSLP after the binding of shortlisted compounds, we calculated the RMSF value over the time. As shown in Figure 5, most residues across all complexes exhibit low RMSF values (close to 0–2 Å), indicating overall structural rigidity and stability in most parts of the protein-ligand complexes. A notable increase in RMSF is observed around residues 110–130, indicating that these residues experience greater flexibility or fluctuations, potentially due to loop regions, termini, or binding interactions that are more dynamic (Figure 5a). Detailed examination of the 3D structures obtained at different simulation time points (50 ns, 100 ns, 150 ns, and 200 ns) revealed that the fluctuations primarily occurred in loop regions. By comparing the peaks 1, 2, and 3 with the 3D structure of TSLP, we found that these are loop regions (Figure 5b–e). This finding indicates that the observed changes are likely a result of the natural flexibility in these loop segments, which may play a role in the dynamic behavior of the shortlisted compound-TSLP interaction.

**Figure 6:**
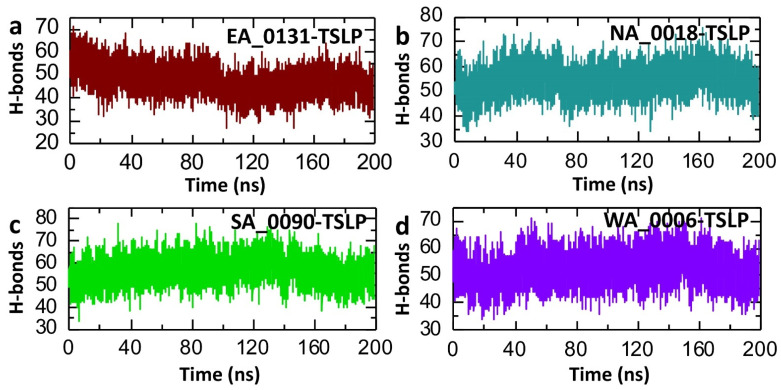
Post-simulation hydrogen bond analysis of shortlisted compound-TSLP complexes. (**a**) represents the post-simulation trajectories of average hydrogen bonds in the EA_0131-TSLP complex. (**b**) represents the post-simulation trajectories of average hydrogen bonds in the NA_0018-TSLP complex. (**c**) represents the post-simulation trajectories of average hydrogen bonds in the SA_0090-TSLP complex. (**d**) represents the post-simulation trajectories of average hydrogen bonds in the WA_0006-TSLP complex.

The hydrogen bond (H-bond) analysis provides information on the stability and interactions within a protein–ligand complex over the course of a molecular dynamics (MD) simulation [55]. H-bonds play a crucial role in maintaining the structural integrity and stability of protein–ligand complexes, and a higher number of stable H-bonds generally suggests a more robust and tightly bound complex [56]. Consequently, to check the binding stability of our shortlisted compounds with the TSLP target, we calculated the post-simulation average hydrogen bonds for each complex. The H-bond count for EA_0131-TSLP starts relatively high, around 60–70, indicating strong initial interactions within the complex. Over time, the number of H-bonds gradually decreases, stabilizing between 40 and 50 H-bonds after about 50 ns and maintaining this range throughout the remainder of the simulation (Figure 6a). The decrease in H-bonds over time suggests that EA_0131-TSLP may undergo some structural adjustments, potentially impacting its stability. This aligns with the observations from the RMSD and Rg analyses, which indicated moderate flexibility and expansion in certain regions. However, NA_0018-TSLP, SA_0090-TSLP, and WA_0006-TSLP showed a relatively similar pattern with an average number of hydrogen bonds of 54, 56, and 53, respectively (Figure 6b–d). This analysis revealed the strong binding of the shortlisted compounds with TSLP and also further verify the RMSD and Rg results. 

### 3.5. Binding Free Energies Calculation

The MMGBSA (molecular mechanics/generalized Born surface area) approach is crucial in drug–protein studies for predicting binding affinities and understanding binding mechanisms. By calculating binding free energies, MMGBSA helps quantify the strength of interactions between a drug and its protein target, which is essential in assessing potential drug efficacy [57,58]. It also offers a more accurate ranking of drug candidates than docking scores by incorporating molecular mechanics and solvation effects. Additionally, MMGBSA decomposes binding energy into components, revealing detailed insights into the forces driving interactions. This method balances accuracy and computational efficiency, making it an effective tool in virtual screening and drug optimization. The binding free energy calculations presented in Table 2 provide insights into the interaction stability between the TSLP protein and four compounds (EA_0131, NA_0018, SA_0090, and WA_0006) using MM/GBSA and MM/PBSA methods. In MM/GBSA analysis, the contribution of van der Waals energies for EA_0131, NA_0018, SA_0090, and WA_0006 complexes were recorded to be −49.2608, −42.7944, −33.0569, and −56.1235 kcal/mol, respectively. Similarly, the recorded electrostatic energies were −198.258, −203.6055, −174.1935, and −191.6528 kcal/mol. However, the total binding free energies were −40.5602, −41.0967, −27.3293, and −51.3496 kcal/mol, respectively (Table 2). Furthermore, in MM/PBSA analysis, the total binding free energies for EA_0131, NA_0018, SA_0090, and WA_0006 complexes were −33.0122, −33.4086, −27.2503, −39.3363 kcal/mol (Table 2). The van der Waals energy shows significant contributions across all compounds, with WA_0006 displaying the strongest interaction at −56.1235 kcal/mol in MM/GBSA, indicating favorable hydrophobic interactions. Electrostatic energy values are also notably high, with NA_0018 exhibiting the highest value at −203.6055 kcal/mol, suggesting strong electrostatic binding. Overall, the total free energy indicates WA_0006 as the most stable complex in both MM/GBSA (−51.3496 kcal/mol) and MM/PBSA (−39.3363 kcal/mol) calculations, which further validates the molecular simulation results. 

### 3.6. Pharmacokinetics Analysis of Shortlisted Compounds

Pharmacokinetics (PK) analysis is crucial for understanding how drugs are absorbed, distributed, metabolized, and excreted within the body, which directly impacts their efficacy and safety [59]. By examining these processes, PK analysis helps optimize dosing, minimize side effects, and prevent harmful drug interactions, making it essential for developing safe, effective medications. It also supports personalized medicine by adjusting dosages based on individual patient factors like genetics or organ function. PK insights are vital for regulatory approval and guide drug formulation to improve bioavailability, ultimately enhancing therapeutic outcomes and patient safety [60]. The pharmacokinetic analysis of selected compounds provides insights into their potential as orally bioavailable drugs and their behavior within the human body [61]. All compounds (EA_0131-TSLP, NA_0018-TSLP, SA_0090-TSLP, and WA_0006-TSLP) comply with Lipinski’s rule of five, indicating they are likely to be orally active, with no violations in molecular weight, hydrogen bond donors and acceptors, or lipophilicity (Log P). Bioavailability scores vary, with EA_0131-TSLP, NA_0018-TSLP, and WA_0006-TSLP displaying higher predicted bioavailability (0.55) compared to SA_0090-TSLP (0.11) (Table 3). This score is an estimation of how much of the drug enters the bloodstream when administered. High bioavailability suggests the drug will reach systemic circulation effectively, an important factor in determining dosage and efficacy. 

Examining additional pharmacokinetic properties, EA_0131-TSLP exhibits low water solubility (Log S = −6.543), whereas SA_0090-TSLP is more soluble (−2.886). While all compounds but SA_0090-TSLP have high predicted human intestinal absorption, only WA_0006-TSLP has the highest volume of distribution (VDss = 0.72), suggesting it may achieve broader tissue distribution. Inhibiting enzymes like CYP450 1A2 indicates potential for drug interactions, as these enzymes are involved in drug metabolism. Compounds that inhibit CYP enzymes may alter the metabolism of co-administered drugs, which is a critical consideration in multi-drug regimens to avoid adverse effects [62]. Our analysis revealed that all of the shortlisted compounds have no inhibitory effect on CYP450 1A2. None of the compounds show permeability across the blood–brain barrier, limiting potential central nervous system effects. All compounds are predicted to be non-toxic with no AMES toxicity, hepatotoxicity, or skin sensitization, presenting a favorable safety profile (Table 4). These combined characteristics highlight each compound’s unique advantages and limitations for further consideration in drug development.

## 4. Conclusions

Our study highlights the potential of natural compounds targeting thymic stromal lymphopoietin (TSLP) as promising therapeutic agents for managing atopic dermatitis (AD) and related inflammatory conditions. Using computational approaches, we identified four compounds with strong binding affinity and stability with TSLP, underscoring the importance of leveraging natural sources to discover accessible, low-toxicity treatments for chronic inflammatory diseases. These compounds demonstrated the capacity to modulate TSLP-driven inflammatory pathways, providing a solid rationale for their therapeutic potential in relieving AD symptoms. The computational methodology employed in this study, integrating molecular docking, molecular dynamics (MD) simulations, and pharmacokinetic evaluations (ADMET), is versatile and adaptable to diverse compound libraries, including synthetic and geographically diverse natural products. This flexibility broadens the model’s relevance to various populations and enables applications beyond TSLP to other critical cytokines, such as IL-33 and IL-25, which are implicated in type 2 inflammatory responses. By targeting multiple cytokine-driven pathways, this approach has the potential to identify compounds with broad therapeutic effects. Importantly, the model can incorporate genetic variability observed across populations, allowing for tailored drug screening to address population-specific therapeutic needs. This customization supports the development of precision medicine approaches for AD and other inflammatory disorders. Moreover, the framework is adaptable for evaluating multi-target therapies, identifying compounds capable of synergistically interacting with multiple biological pathways—a significant advantage for managing complex diseases involving cytokine interplay, immune cell activation, and epithelial barrier dysfunction. While the findings are promising, further preclinical and clinical studies are essential to validate these computational predictions and optimize the selected compounds’ structures for therapeutic use. Such studies will address the potential regulatory requirements for translating these compounds into viable therapies. Regulatory pathways must ensure that the safety, efficacy, and pharmacokinetics of these natural compounds meet international standards. Ethical considerations include respecting biodiversity and traditional knowledge by adhering to fair bioprospecting practices and ensuring equitable benefit-sharing with the communities from which these compounds are derived. Ultimately, this study not only advances our understanding of TSLP-driven pathogenesis but also demonstrates the broader applicability of the computational model to other inflammatory and allergic conditions. By expanding its scope to include diverse biological targets and populations, this integrative approach provides a robust platform for global drug discovery and precision medicine. It opens new avenues for natural-compound-based interventions, offering sustainable and ethically sound solutions for managing chronic diseases.

## Figures and Tables

**Table 1:** List of top hit compounds along with structures and docking scores.

Drug ID	Two-Dimensional Structure	Name	Docking Score
SA_0090	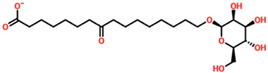	8-oxo-16-[(2R,3S,4S,5S,6R)-3,4,5-trihydroxy-6-(hydroxymethyl)tetrahydropyran-2-yl]oxy-hexadecanoic	−7.37 kcal/mol
EA_0131	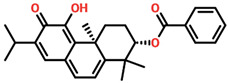	[(2S,4aS)-5-hydroxy-7-isopropyl-1,1,4a-trimethyl-6-oxo-3,4-dihydro-2H-phenanthren-2-yl]	−7.10 kcal/mol
NA_0018	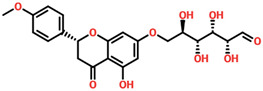	(2R,3S,4R,5R)-2,3,4,5-tetrahydroxy-6-[(2R)-5-hydroxy-2-(4-methoxyphenyl)-4-oxo-chroman-7-yl]oxy-hexa	−7.03 kcal/mol
WA_0006	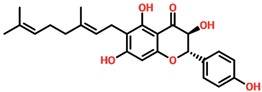	(2S,3S)-6-[(2E)-3,7-dimethylocta-2,6-dienyl]-3,5,7-trihydroxy-2-(4-hydroxyphenyl)chroman-4-one	−6.99 kcal/mol

**Table 2:** List of binding free energies calculated for shortlisted compounds by using MM/GBSA.

MM/GBSA				
Parameters	EA_0131-TSLP	NA_0018-TSLP	SA_0090-TSLP	WA_0006-TSLP
ΔEvdw	−49.2608	−42.7944	−33.0569	−56.1235
ΔEele	−198.258	−203.6055	−174.1935	−191.6528
EGB	212.2015	210.2378	184.1764	202.0759
ESURF	−5.2429	−4.9346	−4.2553	−5.6492
Delta G Gas	−247.5188	−246.3999	−207.2504	−247.7763
Delta G Solv	206.9586	205.3033	179.9211	196.4268
∆G total	−40.5602	−41.0967	−27.3293	−51.3496
MM/PBSA				
Parameters	EA_0131–TSLP	NA_0018–TSLP	SA_0090–TSLP	WA_0006–TSLP
vdW	−49.2608	−42.7944	−33.0569	−56.1235
EEL	−198.258	−203.6055	−174.1935	−191.6528
EPB	218.3227	216.6539	183.0231	212.3594
ENPOLAR	−3.8162	−3.6625	−3.023	−3.9193
DELTA G gas	−247.5188	−246.3999	−207.2504	−247.7763
DELTA G solv	214.5066	212.9913	180.0001	208.4401
DELTA TOTAL	−33.0122	−33.4086	−27.2503	−39.3363

**Table 3:** Lipinski’s rule of five analysis of shortlisted compounds.

Drug ID	Molecular Weight	Hydrogen Acceptors	Hydrogen Donors	Consensus Log P	Lipinski’s Rule		Bioavailability
					Results	Violation	
EA_0131-TSLP	418.5	4	1	5.88	Yes	0	0.55
NA_0018-TSLP	448.42	10	5	0.13	Yes	0	0.55
SA_0090-TSLP	447.54	9	4	0.19	Yes	0	0.11
WA_0006-TSLP	424.49	6	4	4.71	Yes	0	0.55

**Table 4:** List of pharmacokinetic properties of shortlisted compounds.

Drug ID	Water Solubility Log S	Human Intestinal Absorption (%)	VDss (Human)	BBB Permeability	CYP450 1A2 Inhibitor	AMES Toxicity	Skin Sensitization	Hepatotoxicity
EA_0131-TSLP	−6.543	High	0.364	No	No	No	No	No
NA_0018-TSLP	−3.349	High	−0.388	No	No	No	No	No
SA_0090-TSLP	−2.886	Low	−1.26	No	No	No	No	No
WA_0006-TSLP	−3.477	High	0.72	No	No	No	No	No

## Data Availability

All data created are shown in the main text.
